# An evidence-based perspective on Lower Urinary Tract Symptoms and telemedicine during the COVID-19 pandemic

**DOI:** 10.1007/s12553-021-00576-0

**Published:** 2021-07-17

**Authors:** Linda Collins, Rajvinder Khasriya, James Malone-Lee

**Affiliations:** 1 Health, Social Care and Education, Kingston University and St George’s University of London, London, UK; 2 Department of Renal Medicine, Division of Medicine, University College London, London, UK

**Keywords:** Telemedicine, LUTS, Urology, COVID-19

## Abstract

The severe acute respiratory syndrome coronavirus-2 (SARS-CoV-2) causing the COVID-19 pandemic, has had an enormous effect on conventional clinical practice. Telemedicine has emerged as critical to the provision of healthcare services when reducing the transmission of COVID-19 among patients, families, and clinicians. It has been an essential tool for continuing care for patients with lower urinary tract symptoms (LUTS) during the COVID-19 pandemic and has been the link between socially distant patient contact. The aim of this perspective paper was to identify the strengths and limitations of technology-based care focusing on literature linked to patients with lower urinary tract symptoms (LUTS). We search PubMed and CINHAL Plus for grey literature and secondary research on LUTS and telemedicine during the COVID-19 pandemic. Publications dated between the year March 2020 and March 2021were searched. We gathered key specialist opinions in the field of LUTS from several countries around the world, including the countries that had been hit significantly with COVID-19. This perspective paper proposes that there is evidence to support the use of modern technology to facilitate continued healthcare services for patients with LUTS during the COVID-19 pandemic. Telemedicine has been recognised a crucial digital tool for diagnosis, treatment and follow-up appointments during a time of social distancing. Although there are many advantages of telemedicine, the older adult population and those economically disadvantaged with technology may not benefit from technology-based healthcare. The available literature on telemedicine during the COVID-19 pandemic has proven to be successful in the management of some patients with LUTS. It is certain that the COVID-19 pandemic has given telemedicine a significant drive for implementation now and in the immediate future. Robust data on long-term efficacy and safety of telemedicine is required to ensure there are governance protocols embedded when looking after patients with LUTS.

## Introduction

The severe acute respiratory syndrome coronavirus-2 (SARS-CoV-2) causing the COVID-19 pandemic [1] disrupted societies all over the world. This has had an enormous effect on conventional clinical practice. Social distancing forced changes in the delivery of care in outpatient services so as to balance provision against while minimising risk of viral transmission to patients and health care professionals [2]. In these circumstances there is always a risk that what are perceived to be less pressing needs, such as the treatment of lower urinary tract symptoms (LUTS) and incontinence may be subordinated to more concerning problems such as cardiac disease with a COVID-19 diagnosis [3] or cancer treatment with adverse COVID-19 symptoms [4], as well as pre-existing respiratory diseases such as COPD in addition to COVID-19 [5]. This may affect patients and staff alike who will tend to weigh risk against need so that those with chronic diseases are not disadvantaged. Decisions on the distribution of resources will also tend to favour those sectors dealing with SARS-CoV-2 infection [6]. It is important that the clinicians working in the relevant specialities act to protect the interests of vulnerable patients who may be disadvantaged by these influences. One approach it to use technological advances to provide consultations that are safe. Real-time videoconferencing and telephone consultations, collectively termed telemedicine [7] are obvious options. Telemedicine is defined as a technology enabled care service providing care for patients with long term conditions that is convenient, accessible and cost-effective [8]. According to Calton et al. [9] telemedicine has emerged as critical to the provision of medical care when reducing the transmission of COVID-19 among patients, families, and clinicians. A digital patient is defined as a patient who uses and engages with mobile health technologies such as telemedicine [10]. Although there have been studies that have evaluated the effectiveness of telemedicine for service improvement [11–13], telemedicine is yet to be evaluated from the perspective of older adults with LUTS receiving care during the COVID-19 pandemic. The symptoms are debilitating but not life-threatening and may be triaged out of face-to-face provision. The aim of this evidence-based perspective was to identify the strengths and limitations of technology-based care and the future of telemedicine focusing on literature linked to patients with lower urinary tract symptoms (LUTS).

## Methodology

We gathered key specialist opinions in the field of LUTS from several countries around the world, including the countries that had been hit significantly with COVID-19. The research questions, search procedure and inclusion and exclusion criteria are discussed below. This evidence-based perspective was guided by the following questions, what are the strengths and limitations of technology-based continuity of care for patients with lower urinary tract symptoms (LUTS)? What is the role of LUTS telemedicine in the future? What are the recommendations for quality assurance for long term use of telemedicine?

The evidence-based perspective was built using PubMed and CINHAL Plus databases. We decided to select all papers evaluating the use of telemedicine for patients diagnosed with LUTS. An electronic table (Table 1) was created by one of the authors to insert a list of the selected papers for outcome data, strengths, limitations and the future use of telemedicine for LUTS. The electronic table was double checked by the second author for accuracy, relevance and the risk of data bias using the ROBIS tool to assess risk of bias in systematic reviews [14]. Three authors reviewed the titles and abstracts of the records listed in each of the databases and selected the papers that were relevant to this evidence-based perspective. The papers were selected based upon their significance and were assessed in full text to review the findings.

**Table 1:** Included Studies

Author/Year study published/Reference	Country	Study design	Outcome	Strengths and limitations of technology-based care
Grimes et al. [15] A guide for urogynecologic patient care utilizing telemedicine during the COVID-19 pandemic: review of existing evidence. Int Urogynecol J. 27: 1–27	USA	Systematic review and Census study	A critical element of transitioning to telemedicine is maintaining the unique elements of trust, privacy and information-sharing that occur between provider and patient	Strength: Telemedicine may be effectively used for providing a preliminary assessment of new patients and to assist in the follow-up of uncomplicated established patients Limitations: Limited internet access and technical capabilities for some elderly patients may hinder telemedicine follow-up
López-Fando et al. [17] Management of Female and Functional Urology Patients During the COVID Pandemic. Eur Urol Focus 6(5): 1049–1057	Belgium Brazil France Italy Portugal Spain Turkey United Kingdom USA	International narrative review	Efforts should be made to minimize the burden for this patient group, without endangering patients and health care workers during the COVID-19 pandemic	Strength: Telemedicine is a method for follow-up cases not requiring a physical examination or other testing methodologies Limitations: Telemedicine may also not be available to some patients or they may not be able to use technological devices (e.g., older and economically disadvantaged populations)
Medina-Polo et al. [16] Benign prostatic hyperplasia management during COVID-19 pandemia. Arch Esp Urol 73(5): 405–412	Spain	Comprehensive review of the literature	The diagnosis and prescription of treatment for LUTS and BPH during the COVID-19 pandemic should be based on telemedicine and joint protocols for primary care attention and urology	Strength: Promotion of telemedicine and joint protocols for Primary Care Limitations: Clear diagnostics, treatment criteria and referral could be limited
Novara et al. [18] Telehealth in Urology: A Systematic Review of the Literature. How Much Can Telemedicine Be Useful During and After the COVID-19 Pandemic? Eur Urol 78(6): 786–811	Italy	Systematic review on Telehealth and Urological applications	Telemedicine has been implemented successfully in several common clinical scenarios, such as follow-up care, of uncomplicated urinary stones and uncomplicated UTIs	Strength: Telehealth is successful for uncomplicated LUTS patients Limitation: Safety concerns for patients with malignant or complex diagnoses
Somani et al. [19] Delivery of urological services (telemedicine and urgent surgery) during COVID-19 lockdown: experience and lessons learnt from a university hospital in United Kingdom. Scott Med J 65(4): 109–111	United Kingdom	7-week observation of patients attending an outpatient department for LUTS during a national lockdown	COVID-19 virtual outpatient clinics with telemedicine for patients with LUTS and urgent urological surgery can continue to be carried out to minimise disruption to services	Strength: Potentially mitigate the impact of delaying patients most in need Limitation: Risk of losing of vital clinical information

### Inclusion and exclusion criteria

The main search terms used in both databases were telemedicine, LUTS, Urology and COVID-19 and we searched for grey literature and secondary research dated between the year March 2020 and March 2021. The inclusion criteria included peer reviewed papers, with access to full text and related to telemedicine, LUTS, Urology and COVID-19. Studies not linked to the inclusion criteria or focused on the related questions were excluded.

## Results

**Fig. 1:**
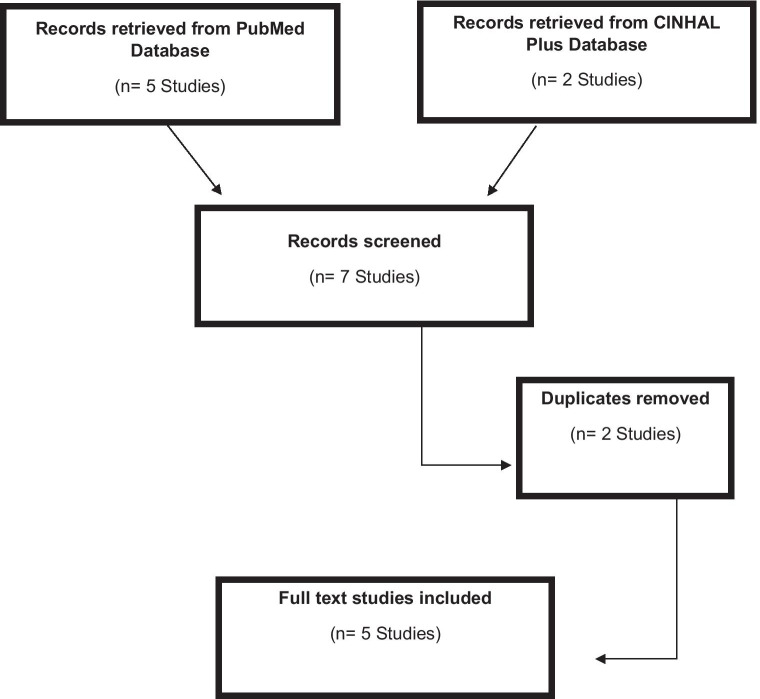
Literature retrieval flow diagram

Limited records were retrieved based upon the publication date and the nature of the topic. A total of five records were retrieved from PubMed and two from CINAHL Plus. The two papers retrieved from CINHAL Plus were duplicates found in the PubMed search and were removed. The remaining four papers were selected as they were relevant to “telemedicine”, “LUTS”, “Urology” and “COVID-19” and provided data for our perspective. The process of literature retrieval and selection has been illustrated in a flow diagram (Fig. 1) which complied with the framework for reporting narrative synthesis [11]. Of the five papers included within this evidence-based perspective, four were review papers [15–18] and one was an observational study [19]. The authors who published the review papers evaluated existing literature on the effectiveness of telemedicine for patients with LUTs and indicated that telemedicine had been implemented successfully in several clinical scenarios during the pandemic. Out of the four review papers, one paper was an international narrative review of existing literature, with collaboration from leaders in the field of female and functional urology (FFU) from several countries around the world. Their goal was to develop a strategy that reorganises FFU activity, diagnosis and treatment during the COVID-19 pandemic based upon technology through the synthesis of evidence. The authors from the observational study examined their clinical activity and the use of telemedicine during a 7-week COVID-19 lockdown as part of departmental planning for continued urological services. Their findings highlighted the importance of flexible healthcare delivery with the use of telemedicine during the pandemic and in the future.

The COVID-19 pandemic has shown that telemedicine is a pertinent and accessible method for delivering efficient healthcare for patients and clinicians [20]. A recent study has shown a large proportion of patients are willing to keep a telemedical appointments scheduled during the COVID-19 pandemic [21]. The advantage of being able to attend a follow-up appointment without attending a clinical unit has been recognised as an advancement in healthcare service delivery and mitigates the impact of delayed follow-up appointments for patients who require subsequent reviews [19]. López-Fando et al. [17] highlighted that telemedicine was an essential method for patient follow-up during the COVID-19 pandemic particularly for LUTS cases not requiring a physical examination or other testing methodologies. Medina-Polo et al. [16] also identified the advancement of telemedicine during the pandemic but were aware that telemedicine promoted the need for joint implemented policies for patients with LUTS in primary care. Although telemedicine has existed prior to COVID-19 in LUTS services, the recognition of it being an essential part of healthcare service delivery is merited. The LUTS clinic at Whittington Health North London, implemented telemedicine from the year 2010, when the patient numbers grew beyond local tertiary referrals. It was evident then that LUTS and telemedicine was the way forward for managing patients nationally. Novara et al. [18] indicates that telehealth is successful for uncomplicated LUTS patients however, complex patients with chronic urinary tract infections (UTI) have been successfully managed by telemedicine supplemented by urine specimen examinations [22] which can be achieved at different times and close to the patients’ locales. Somani et al. [19] have recognised through their observational study, that the COVID-19 pandemic has encouraged a greater openness to virtual outpatient services providing healthcare that minimises disruption to continuing care. Similarly, Grimes et al. [15] have laid emphasis on the multidimensional qualities of telemedicine, describing how the pandemic has forced a shift in how patients can receive continued, seamless care in more convenient and efficient way with high levels of patient satisfaction.

The publications have highlighted the fact that telemedicine may not be accessible to all, such as some in the older adult population, individuals who are economically disadvantaged, persons disadvantaged by language, cognition and technophobia although families and friends can help much with these [15, 17]. Such persons are at risk of falling through the net, being lost to follow-up or harmed through information deficiencies [19]. This is particularly the case for patients with complex diagnoses [18] and LUTS hosts an increased representation of these groups. Some people buck the social trends and experience shyness and inhibitions when discussing personal matters across an electronic connection. With the advancements in health technologies and the move towards integrated healthcare management systems, there is a need to ensure that patients with LUTS are abreast of these technological changes. Healthcare professionals have a vital role when advocating the use of health technology, but more importantly have a duty of care to follow up with patients who lack digital literacy and adopt minimal use of digital apps and electronic devices for their healthcare management.

## Discussion

### The future of LUTS telemedicine post COVID-19

During the COVID-19 pandemic, Medina-Polo et al. [16] believed that the diagnosis and treatment for patients with LUTS should be based on telemedicine. Novara et al. [18] have identified that the future of telemedicine can be usefully implemented to guide patients in the decision-making process, diagnosis of LUTS and treatment selection for individual cases. Somani et al. [19] 7-week observational study highlighted that the use of telemedicine had risen steeply during the COVID-19 pandemic and is likely to become a sustainable reality post pandemic. The acceptance and integration of telemedicine in Urogynecology practice for patients with LUTS is possible, due to rapid advances in telecommunications and digital technology [15]. Patients with chronic UTI have relied on telemedicine and empirical antibiotic therapy as an effective method of treating the condition and lowering clinical pressure on health services [23]. It would seem that telemedicine will be playing a greater part healthcare service delivery as it is a practice that has currently expanded beyond traditional diagnostic and monitoring activities [18]. This is a good advancement, but López-Fando et al. [17] highlights the need to tread carefully into the world of telemedicine by working to minimise the special risks that will affect subsets of our patient population.

Telemedicine should continue to be used if it minimises disruption to services and patient care [19]. Transitioning to telemedicine for managing LUTS and pelvic floor disorders is critical for continuing conservative treatment [15]. Sacco et al. [24] acknowledge that virtual consultations providing clinical instructions and telemonitoring of patient symptoms is the way forward, but they lay emphasis on the importance of patient assessment, engagement and evaluation of telemedicine efficacy for long-term use. There is no doubt that telemedicine has contributed to efficient service provision, and in the case of patients with LUTS, has been an essential tool for patient contact when social distancing had been a major factor. Relying on face-to-face consultations as the main source of service provision may soon be recognised as past practice, as contemporary management of LUTS patients through digital technology is widely used. Somani, Pietropaolo et al. believe that clinicians and healthcare professionals should be trained on how to effectively use telemedicine prior to mass implementation, to maximise healthcare delivery but without compromising patient safety [19]. It is undeniable that healthcare professionals are relied upon to maintain patient safety and provide quality care. Telemedicine has a multifaceted approach, enabling care provision for patients with long term conditions that is convenient, accessible and economically sustainable [8]. As Somani et al. have mentioned, training is essential to maximise effective use of telemedicine, but the emphasis on patient safety outweighs mass implementation, and education, training and further professional development are significant factors for maximising the use of telemedicine.

## Recommendations for quality assurance for long term use of telemedicine

There is published evidence that telemedicine has been implemented successfully in the management of uncomplicated LUTS [18]. The recommendations from Medina-Polo et al. literature review [16] suggests that the management of LUTS-Benign prostatic hyperplasia during and after the COVID-19 pandemic should consist of telemedicine and joint protocols for delivering telemedicine within Primary Care. It would be wise to ensure that proper feedback loops are established at this early stage so that our enthusiasm is not allowed to neglect unknown and unforeseen adverse consequences that nobody imagined. The findings from Novara et al. systematic review supports the concept of robust data collection on long-term efficacy, safety, and health economics, necessary for long term use of telemedicine for patients with LUTS [18]. Somani et al. recommend telemedicine for consultations as implemented within their observational study, recognising it as a useful method to prioritise the patients most in need of urgent care and to maximise a flexible healthcare service delivery [19]. Further understanding of LUTS and telemedicine is continually being uncovered, and patient experience of LUTS and telemedicine during the COVID-19 pandemic is imminent. With new evidence emerging, policy and guidelines on LUTS and telemedicine is needed to ensure that equitable digital care is accessible to all through the national promoted technology enabled care services [8]. Further research is needed to quantify the impact of LUTS telemedicine in comparison to face-to-face consultations and the advantages and disadvantages of its long-term use.

## Conclusion

We do not know whether COVID-19 will be present for the years to come. Nevertheless, we should be prepared to provide efficient healthcare service throughout. The available literature on telemedicine during the COVID-19 pandemic has provided encouragement for the future use of these methods for the care of patients with LUTS. It would see that some complex bladder conditions are suitable for telehealth. We are optimistic about the future potential but emphasise the importance of building in thorough safety surveillance feedback systems that ensure quality assurance for clinicians and health care professionals as well as patients receiving telehealth services.

## Data Availability

The secondary data used and/or analysed for this perspective paper are available and accessible from the PubMed and CINAHL Plus.
